# A brief but comprehensive three-item social connectedness screener for use in social risk assessment tools

**DOI:** 10.1371/journal.pone.0307107

**Published:** 2024-07-19

**Authors:** Nancy P. Gordon, Matthiew C. Stiefel

**Affiliations:** 1 Division of Research, Kaiser Permanente Northern California, Oakland, California, United States of America; 2 Institute for Healthcare Improvement, Boston, Massachusetts, United States of America; University of Nottingham Ningbo China, CHINA

## Abstract

**Background:**

The 2014 IOM report “Capturing Social and Behavioral Domains and Measures in Electronic Health Records” described three subdomains of social relationships that affect patient health and well-being. However, most social risk screeners currently assess only one subdomain, frequency of social connections. We are proposing a three-item Brief Social Connectedness (SC) screener that additionally assesses risks in social/emotional support and loneliness/social isolation subdomains.

**Methods:**

For this cross-sectional study, we used data from a 2021 Kaiser Permanente Northern California (KPNC) social risk survey for 2244 members ages 35–85 years. The survey included three validated questions that covered the SC subdomains (frequencies of social contacts with people they care about, feeling lonely/socially isolated, and getting enough social/emotional support). Variables representing moderate/high versus low risk were created for each subdomain. We used weighted data for bivariate analyses and modified log-Poisson regression models that adjusted for age, sex, race, and ethnicity to examine cross-sectional associations among the three subdomain risks, as well as with two structural SC risks, living alone and not being in a committed relationship. We then used modified log-Poisson regression models to study cross-sectional associations of these five SC variables with three single-item self-report measures of emotional health.

**Results:**

In regression models that included all five SC variables, loneliness/social isolation and social/emotional support risks were significantly associated with all three emotional health measures, while frequency of social contacts, living alone, and no committed relationship were not. However, low frequency of social contacts and no committed relationship significantly increased risk of often feeling lonely/socially isolated and lacking in social/emotional support.

**Conclusions:**

A three-item social connectedness screener that assessed risks of loneliness/social isolation, inadequate social/emotional support, and low frequency of social contacts provided more comprehensive information about emotional health risks than social connection frequency alone.

## Introduction

There is extensive evidence that social connectedness–the degree to which people have and perceive a desired number, quality, and diversity of relationships that create a sense of belonging, and being cared for, valued, and supported [1]–is a significant social determinant of health and emotional well-being [2, 3]. In their 2014 report *Capturing Social and Behavioral Domains and Measures in Electronic Health Records*, the Institute of Medicine (IOM) Subcommittee on Social and Behavioral Determinants of Health (SBDoH) described three subdomains of social connectedness (SC) that affect patient health and well-being and use of and need for healthcare services [4]

- Social integration (or its converse, lack of social contacts and having few people to interact with regularly);
- Perceived level of social and emotional support obtained from other people; and
- Extent to which the individual feels socially isolated or lonely.

The IOM report suggested that information about how their patients are faring in all three SC subdomains could help healthcare providers better understand their patients’ health, health care needs, and patterns of healthcare utilization. Additionally, systematic screening for deficits in any of these SC subdomains could help healthcare systems identify and intervene with patients at elevated risk for poor physical and emotional health outcomes and undesirable patterns of healthcare utilization [5].

### Problems with the current IOM-recommended screener for social connectedness

To screen for risk in the SC domain, the IOM Subcommittee on SBDoH recommended a version of the Berkman-Syme Social Connection Index (B-S SCI) [6] (See **S1 Appendix**). The B-S SCI, developed in the 1970s, only assesses a single dimension of social relationships (social integration), is lengthy (6 questions), and is out of date in several respects. First, two of the items in this version of the B-S SCI ask about frequency of attending church or religious services and whether the individual belongs to a club or organization. Church and club/organization attendance can now occur (and during the COVID-19 emergency, mostly occurred) via Zoom, streaming services, television, and other remote access channels, whereas when the B-S SCI was developed, attendance was mostly in person. Second, the marital status/partner relationship indicator is limited to people who are married or living together, whereas it is not uncommon now for people to be in a committed relationship while maintaining separate households. Third, as currently scored, a person need only indicate that they belong to a club or organization to get credit for that type of social connection, not that they attend meetings > 4 times a year or participate in person [7].

Perhaps a more major problem with using the B-S SCI to assess social connectedness is that objective and subjective assessments of an individual’s social integration/social isolation and social support can differ [8–10]. The extent to which an individual is socially integrated or socially isolated is usually defined by objective and quantitative measures such as the size and diversity of an individual’s social network and the frequency of social contacts with friends and relatives, attendance of religious services and club/organizational meetings, being in a committed relationship, and living situation (alone or with others) [3, 11]. Similarly, it is possible to objectively measure the amount and types of social support (e.g., emotional or affective, instrumental or tangible assistance, and companionship) that an individual *receives* from people in their social network on an ongoing or as-needed basis. However, *perceived* quality and sufficiency of social and emotional support received from one’s social network, as well as extent to which the individual feels socially isolated or lonely, are *subjective* assessments made by an individual that must be ascertained by directly asking the individual or a close proxy.

### Subdomains of social connectedness

Research has shown that *frequency of social interactions* is not always associated with positive health and mental health outcomes. For example, frequent *negative* interactions with people in one’s social network, especially close relationships like spouse/partner, family members, and co-workers, can lead to depression, anxiety, psychological distress, and unhealthy coping behaviors, which in turn increase risk for adverse health and mental health events [11, 12]. Additionally, the association of frequency of social connection with feelings of loneliness and psychological well-being is influenced by an individual’s perceptions of whether their interactions are providing them with the amount, quality, and types of emotional connection and support they want [11, 13]. Among older adults, for example, research suggests that the number of interactions is not as important as the quality of the interactions and the amount of social support they provide [14, 15].

*Loneliness* has been described as “self-perceived lack of social support and companionship” [10] and as the “discrepancy between one’s desired relationships and one’s actual relationships” that is distinct from actual or perceived social isolation [16–18]. While social isolation is a known risk factor for loneliness [19] and the two conditions often co-occur, people can be socially isolated but not lonely and lonely but not socially isolated [20, 21]. For example, people who are married or not living alone have reported feeling lonely, even though objectively they are not socially isolated [16, 21]. Loneliness has been associated with many adverse health outcomes [22, 23], including increased risk of depression [10, 21], high systolic blood pressure [24], incident coronary heart disease and stroke [25, 26], poorer sleep efficiency [27], cognitive decline [28–30], dementia [30, 31], and premature death [32].

*Perceived social support* has been linked to mental well-being [33, 34]. Adequacy of perceived emotional support has been shown to play a significant role in protecting individuals from developing depression [35, 36] and psychological distress [37] when faced with stressful life events. Level of perceived social support has also been associated with psychological adjustment to acute and chronic health conditions such as HIV [38], rheumatoid arthritis [39], cancer [40–42], and cardiovascular disease [43]. Further, level of perceived emotional support, especially from friends, has been shown to be negatively correlated with loneliness [44, 45].

### Need for assessment of all three subdomains of social connectedness

In a recent overview of social connection as a public health issue, Holt-Lunstad suggests that the modest correlations between objectively measured social connectedness, loneliness/perceived social isolation, and perceived social support found in previous research reinforce that these are related but distinct domains that contribute to physical and emotional health and well-being in unique ways. Furthermore, none of these domains alone can adequately capture assess risk related to deficits in social connectedness and social support [3].

In this article, we propose a replacement for the single domain B-S SCI that uses 3 validated questions to cover all three of the IOM Subcommittee-specified SC subdomains. Our study had three specific aims. First, we wanted to examine the inter-relationships of the SC subdomain risks derived from the proposed 3 screening questions to assess the extent to which they represent distinct subdomains of SC. Second, we wanted to assess how well the three screening items performed individually and together in identifying adults who were experiencing emotional troubles. Third, we wanted to learn whether adding information about two frequently assessed structural SC measures, being in a committed relationship and living situation (alone versus with other people), led to a meaningful improvement in ability to identify adults who were experiencing emotional stress beyond use of only the three SC subdomain variables such that they should be considered as valuable additional items for SC screening.

## Methods

### Study design and setting

For this cross-sectional study, we analyzed data from an English-only self-administered (mailed print or online) social risk survey that was conducted with a stratified random sample of middle-aged and older adult Kaiser Permanente Northern California (KPNC) health plan members who did not have health insurance through Medi-Cal (California’s Medicaid government insurance program for very low-income adults). KPNC is a healthcare delivery system that provides integrated primary and specialty care to > 4 million health plan members who mostly reside in the San Francisco Bay Area and greater Sacramento Valley and Central Valley areas of California. The KPNC adult membership has been shown to be very similar to the insured population of Northern California adults with regard to health and sociodemographic characteristics, with the exception of having smaller percentages of very low income and Medi-Cal covered adults [46, 47].

### Data source and study population

The KPNC Social Risk Survey was fielded from April 14, 2021 to December 7, 2021, approximately one-year into the COVID-19 emergency. More information about and results from the survey are found in an earlier publication [48]. For this study, we restricted analyses to 2244 (out of 2869) respondents aged 35–85 years who answered all three SC screener items. The sociodemographically diverse sample was comprised of 2244 adults aged 35–85 years (mean 67.6 years, std.11.0) and included 1242 women, 1002 men, and 681 non-Hispanic White, 515 Black, 528 Latino, 505 Asian/Pacific Islander, and 15 Other race/ethnicity. All respondents had sufficient English proficiency to complete the questionnaire in English. To create the analytic study population, respondent data were weighted to the age, sex, racial, and ethnic composition of the 2019 KPNC membership whose preferred written and spoken language in the electronic health record (EHR) was English.

### Ethics

This study was approved by the KPNC Institutional Review Board, which waived the requirements to obtain informed consent as allowed under {§46.116(d)} and Privacy Rule Authorization for use and disclosure of protected health information (PHI) as allowed under {45 CFR 164.512(i)(1)(i)}.

### Study variables

#### Social connectedness screener variables

Our three-item SC screener uses questions taken from two widely used social risk screeners and the National Health Interview Survey. These three questions have each been extensively cognitively and field tested in English and multiple other languages. A response considered to indicate moderate or high risk on any of these three screening questions would suggest that the individual should be further assessed for issues in the SC domain.

- *Social connection frequency* was assessed using the question: “How often do you see or talk to people that you care about and feel close to? (For example, talking to friends on the phone, visiting friends or family, going to church or club meetings),” with possible responses of < 1 day a week, 1 or 2 days a week, 3 or 4 days a week, and ≥ 5 days a week. This single question, taken from the PRAPARE social risk screener [49], covers most of the sources of social connection asked about in the 6-item Berkman-Syme SCI but restricts to contacts with people with whom the individual has a strong emotional connection versus merely social contacts through attendance at clubs, religious services, or activities of daily living. Per the PRAPARE risk scoring recommendations, we considered a frequency of such contacts < 5 days a week to indicate moderate to high risk in this social connection frequency subdomain. However, because we believed that a ≥ 5 days a week cut-point would result in such high percentages of adults screening positive for this risk in the healthcare setting that the question might not be used, we kept social connection (SOCCON) frequency risk as a four-level variable, using ≥ 5 days a week as a reference level indicating low risk.
- *Perceived social and emotional support* was assessed using the question “How often do you get the social and emotional support you need?” This question was used in the 2020 National Health Interview Survey. As was done by National Center for Health Statistics researchers [50], we classified individuals as having risk in in the social/emotional support subdomain if their responses were “never”, “rarely”, or “sometimes” (SOCSUPT risk = 1) versus “usually” or “always” (SOCSUPT risk = 0).
- *Frequent feelings of loneliness or social isolation* was assessed using the question”Overall, how often do you feel lonely or socially isolated?” This is an optional question in the Accountable Health Communities (AHC) Health-Related Social Needs Screening Tool [51] that was slightly adapted from a question used in a survey of middle-aged and older adults conducted in 2018 by AARP, that was in turn adapted from the UCLA Loneliness Scale [52]. Because people were asked to physically isolate from others but not socially isolate from others (i.e., have contact by phone, video chat, and outdoors or socially distanced meetups) during the first year of the COVID-19 emergency, we slightly modified the question wording from frequency of “feeling lonely or isolated from others” to “feeling lonely or socially isolated” with concurrence from the author of the original UCLA Loneliness Scale, Dr. Letitia Anne Peplau (personal communication). We classified individuals as having risk in the loneliness/social isolation subdomain if they indicated that they often or always feel lonely or socially isolated (LONELINESS risk = 1) versus never, rarely, or sometimes feeling this way (LONELINESS risk = 0).

#### Structural social connectedness variables

Respondents were classified as not being in a committed relationship if they had indicated that they were widowed, separated, or single (NO_RELAT = 1) versus married or in a domestic partnership, living with a partner, or in a committed relationship but not living with the partner (NO_RELAT = 0). This variable modifies the married/living with partner factor currently used to calculate B-S SCI risk to be more inclusive of a now more common situation of committed couples maintaining separate residences. Respondents were classified as living alone (LIVALONE = 1) if they had indicated that they “Live alone (not with another person) in your own home (house, apartment, condo, trailer, etc.)” versus in settings with other people (LIVALONE = 0) based on response to a checklist describing various residential and institutional living situations.

#### Self-reported emotional trouble outcome variables

We created three dichotomized variables from responses to validated single-item patient-reported measures of emotional health. The Dartmouth Coop Feelings item asks, “During the past four weeks, how much have you been bothered by emotional troubles such as feeling anxious, irritable, depressed or sad?” [53]. People who answered “quite a bit” or “extremely” (versus not at all, a little, or somewhat) were classified as being “emotionally troubled.” The CAHPS Commercial Adult Survey contains a global self-rated emotional health item “In general, how would you rate your overall mental or emotional health?” [54]. People who answered “fair” or “poor” (versus excellent, very good, or good) were classified as having “fair/poor emotional health.” The measure of chronic stress, “During the past 3 months, how often have you felt very stressed or tense?” was developed for our survey, but a similar item has been used in every KPNC Member Health Survey starting in 1996. People who indicated feeling very stressed “much of the time” or “most of the time” (versus never, a little of the time, or some of the time) were classified as being “chronically stressed.”

#### Sociodemographic and health status variables

Age at time of survey was an interval variable (35–44, 45–54, 55–64, 65–74, 75–85 years) corresponding to the 10-year intervals used to create the survey weighting factor. Respondent sex was coded as male or female based on self-report. Racial and ethnic group (White, Black, Latino, Asian/PI, Other) was assigned based on self-reported survey data, with respondents indicating multiple racial/ethnic groups assigned to one group based on the race or ethnicity indicated in their EHR. Three variables were created only for purposes of describing the study population. Educational attainment was based on the question “What is the highest level of school you completed”. Financial situation was based on response to the question “Thinking about the past 3 months, would you say that at the end of each month you generally ended up with: More than enough money left over, some money left over, just enough to make ends meet, almost enough to make ends meet, or not enough to make ends meet.”[55] Overall health status was based on response to the question “Would you say that, in general, your health is: excellent, very good, good, fair, or poor?” [56].

### Statistical analysis

Respondents were assigned post-stratification survey weights based on the age (10-year intervals) x sex (male, female) x racial and ethnic (White, Black, Latino, Asian/Pacific Islander, Other) composition of the 2019 KPNC adult membership with English spoken language preference. In our analyses, to prevent variance inflation, we then normalized the population weight so that the total N in the weighted sample equaled the total N of the respondent sample while maintaining the age-sex-racial/ethnic composition of the weighted sample.

We first examined the unadjusted bivariate cross-sectional associations among SOCCON frequency, NO_RELAT, and LIVALONE with LONELINESS risk and SOCSUPT risks using cross-tabular analysis with chi-square tests. We next used modified log-Poisson regression models to produce adjusted prevalence ratios with 95% confidence intervals (CI) that described demographics-adjusted bivariate associations between SOCCON frequency, NO_RELAT, and LIVALONE with LONELINESS risk and SOCSUPT risk that adjusted for sex (female versus male), age (10-year interval variable), and racial or ethnic group (Black, Latino, Asian/Pacific Islander, Other versus White). Adjusted prevalence ratios (aPRs) compare the prevalence of the outcome being modeled (e.g., being emotionally troubled) among those with a risk (e.g., inadequate social/emotional support) to those without that risk after adjusting for covariates. The aPR is a more appropriate measure of association than adjusted odds ratios derived from logistic regression models to use when comparing outcomes that are not rare in the population [57]. Finally, to assess the independent associations of SOCCON frequency, NO_RELAT, LIVALONE, and LONELINESS risk or SOCSUPT risk (depending on which of the latter two subjective SC variables was being modeled) with having LONELINESS risk and SOCSUPT risk, we ran “full models” that included four SC variables along with the demographic covariates.

We used the same 3-step approach to examine the cross-sectional associations of the five SC variables with the three emotional health outcome variables (emotionally troubled, fair/poor emotional health, and chronically stressed). A “full model” was used to evaluate whether SOCCON frequency, LONELINESS risk, and SOCSUPT risk were independently associated with these emotional health outcomes after adjusting for other covariates, as well as whether NO_RELAT and LIVALONE risks were independently associated with the emotional health outcomes after controlling for the other three SC factors.

We then explored how well having information about SC risks, when used in combination, accurately identified individuals who were experiencing emotional difficulties, and whether including NO_RELAT and LIVALONE in the model improved the accuracy of classification. The SAS log-Poisson regression (Proc Genmod) procedure does not calculate a model fit statistic, so we used a two-step approach recommended by Wilkins [58] in which logistic regression models were used to create receiver operating characteristic (ROC) curves and calculate areas under the ROC curves (AUC) to compare model fit using the predicted probabilities from the log-Poisson regression models. For each emotional health outcome variable, we compared AUCs for three models that did not include demographic covariates: (1) a model that included SOCCON frequency, LONELINESS risk, and SOCSUPT risk; (2) a model that included SOCCON frequency, LONELINESS risk, SOCSUPT risk, NO_RELAT, and LIVALONE; and (3) a model that included only LONELINESS risk and SOCSUPT risk.

Finally, we ran the same sets of modified log-Poisson regression models for 35–64 yr and 65–85 yr subgroups to evaluate whether the same pattern of associations observed in the full sample was found in middle-aged and older aged groups. To test for statistically significant age group differences in aPRs, we ran log-Poisson regression models with the full sample that included interaction terms for age group.

All analyses used weighted data and were performed using SAS v9.4 (SAS Institute, Cary, NC, 2014). All percentages and aPRs reported in the text are based on weighted data, and all subgroup differences mentioned in the text are significant at p< 0.05.

## Results

The analytic study population had a mean age of 58.3 years (std. dev = 12.8), was 55% female, had a racial and ethnic composition of 57.5% White, 8.1% Black, 13.9% Latino, 20% Asian/Pacific Islander, and 0.5% other, and was fairly well-educated (83% with at least some college) **(Table 1)**. Approximately 27% of adults were experiencing financial strain, with 19% just “making ends meet” and 7% not “making ends meet.” Most (87.5%) adults reported good (40.4%) or very good/excellent (47.5%) health. About one-fourth of adults were not in a committed relationship and 16% lived alone, but because 12% of adults who lived alone were in a committed relationship, only 14% were more at risk on structural SC factors (i.e., lived alone and not in a committed relationship). Approximately 13.8% of adults had seen or talked with people they cared about or felt close to < 1 day/week, 26.3% had done so 1–2 days/week, 25.2% 3–4 days/week, and 34.7% ≥ 5 days/week. Using the < 5 days/week PRAPARE risk threshold, 65.3% of adults would be considered to have a social connection frequency risk. Approximately one-third of adults had a social support risk, 7.7% had a loneliness risk, and 7.1% had both social support and loneliness risks.

**Table 1:** Characteristics of the study population.

Characteristic	Unwtd N	Wtd. %
Age (years)		
Mean [std.]	2244	58.3 [12.8]
35–44	131	21.4%
45–54	176	20.2%
55–64	281	24.4%
65–74	1077	20.5%
75–85	579	13.5%
Sex		
Male	1002	44.9%
Female	1242	55.1%
Race and ethnicity		
White non-Hispanic	681	57.5%
Black	515	8.1%
Latino	528	13.9%
Asian/Pacific Islander	505	20.0%
Other	15	0.5%
Financial situation during prior three months		
Had more than enough money to make ends meet	1629	73.4%
Had just enough money to make ends meet	411	19.3%
Had almost enough or not enough money to make ends meet	171	7.3%
Educational attainment		
≤ High school graduate	99	2.8%
High school graduate/technical/trade school certificate	382	14.2%
Some college/associate degree	774	29.5%
≥ Bachelor’s degree	968	53.5%
Overall rating of health		
Fair/poor	389	12.1%
Good	979	40.4%
Very good or excellent	861	47.5%
In a committed relationship ^a^		
No	674	25.4%
Yes	1557	74.6%
Lives alone in own home		
Yes ^b^	446	16.0%
No	1791	84.0%
Social connection frequency ^c^		
< 1 day/week	304	13.8%
1–2 days/week	542	26.3%
3–4 days/week	588	25.2%
≥ 5 days/week	810	34.7%
Frequency feels lonely or socially isolated		
Often or always	152	7.7%
Never, rarely, or sometimes	2092	92.3%
Frequency gets enough social and emotional support		
Never, rarely, or sometimes	726	35.2%
Usually or always	1518	64.8%
Felt emotionally troubled in past 4 weeks		
Quite a bit/extremely	307	15.5%
Not at all, a little, somewhat	1924	84.5%
Overall rating of emotional or mental health		
Fair or poor	284	12.7%
Excellent, very good, or good	1940	87.3%
How often has felt very stressed or tense during past 3 months		
Much or most of the time	289	17.2%
Never, a little of the time, or some of the time	1943	82.8%

Unwtd. n: Number of respondents who indicated this response; Wtd. %: Percentage of adults in the study sample with this characteristic based on respondent data weighted to the age, sex, and race of the study population^.^

^a^ Married/domestic partnership, living with partner, or in a committed relationship but not living with partner vs. separated, widowed, or single.

^b^ 12% of adults who lived alone were in a committed relationship

^c^ How often person sees or talks to people they care about or feel close to.

### Inter-relationships of social connectedness risks

The distribution of SOCCON frequency and percentages of individuals with different SOCCON frequencies did not show significant differences based on whether adults lived alone versus with others, were in a committed relationship or not, or lived alone without being in a committed relationship versus lived with others or being in a committed relationship.

The prevalence of LONELINESS risk decreased monotonically as SOCCON frequency increased **(Table 2)**. Specifically, among adults with SOCCON frequency < 1 day/week, 1–2 days/week, 3–4 days/week, and ≥ 5 days/week, respectively, the prevalence of LONELINESS risk was 20.8%, 10.5%, 4.6%, and 2.7%. **Table 2** shows that compared to a SOCCON frequency of ≥ 5 days/week, prevalence of LONELINESS risk was significantly higher for those with social contacts <1 day/week and 1–2 days/week, but those with contacts 3–4 days/week did not significantly differ from those with contacts ≥ 5 days/week. Not shown in the table is that the risk of experiencing frequent loneliness was twice as high for individuals with less than one contact day per week compared to those with 1–2 contact days per week (aPR = 2.27 [1.35–3.82]), and also higher for individuals with 1–2 contact days per week compared to those with 3–4 contact days per week (aPR = 1.54 [1.21–1.95]). However, in the full model predicting LONELINESS risk that also included SOCSUPT risk, NO_RELAT, and LIVALONE, the only significant SOCCON frequency comparison that remained statistically significant was the nearly 3-fold increase in LONELINESS risk for contact <1 day/week vs. ≥ 5 days/week (aPR = 2.98 [1.29–6.72]). As is also shown in **Table 2**, in the full model predicting LONELINESS risk, having SOCSUPT risk was associated with a >15-fold higher prevalence (aPR = 15.00 [7.57–29.74]) and NO_RELAT was associated with a nearly three-fold higher prevalence (aPR = 2.78 [1.62–4.80]) compared to not having these risks, but LIVALONE status no longer had a statistically significant independent association with LONELINESS risk.

**Table 2:** Inter-relationship of five social connectedness variables.

Variable	Often feels lonely or socially isolated (Loneliness risk)			Usually does not get enough social and emotional support (SOCSUPT risk)		
	% (95% CI)	Bivariate model ^a^ aPR (95% CI)	Full model ^b^ aPR (95% CI)	% (95% CI)	Bivariate model ^a^ aPR (95% CI)	Full model ^b^ aPR (95% CI)
Social connection frequency ^c^						
< Once a week	20.8% ^d^ (13.3–28.4)	**9.24** **(4.14–20.65)**	**2.98** **(1.29–6.92)**	70.2% ^d^ (62.6–77.7)	**4.32** **(3.27–5.70)**	**3.61** **(2.70–4.82)**
1–2 times/week	10.5% ^d^ (6.7–14.3)	**4.06** **(1.85–8.96)**	1.73 (0.77–3.87)	48.1% ^d^ (41.5–54.7)	**2.90** **(2.17–3.86)**	**2.68** **(2.01–3.57)**
3–4 times/week	4.6% ^d^ (1.6–7.6)	1.79 (0.69–4.62)	1.12 (0.44–2.88)	29.3% ^d^ (23.3–35.3)	**1.77** **(1.28–2.45)**	**1.74** **(1.27–2.39)**
≥ 5 times/week (Ref)	2.6% ^d^ (0.8–4.4)	(Ref)	(Ref)	15.9% (11.9–19.9)	(Ref)	(Ref)
How often gets enough social and emotional support						
Never, rarely, or sometimes	0.9% ^d^ (0.3–1.4)	**23.65** **(12.09–46.28)**	**15.00** **(7.57–29.74)**			
Usually/always (Ref)	20.3% (15.6–24.9)	(Ref)	(Ref)			
How often feels lonely or socially isolated						
Often/always				92.6% ^d^ (88.0–97.3)	**2.98** **(2.62–3.40)**	**1.94** **(1.63–2.31)**
Never, rarely, or sometimes (Ref)				30.4% (27.2–33.6)	(Ref)	(Ref)
In a committed relationship ^e^						
No	16.6% ^d^ (11.8–21.4)	**3.50** **(2.15–5.69)**	**2.78** **(1.62–4.80)**	45.1% ^d^ (38.7–51.5)	**1.48** **(1.25–1.80)**	**1.32** **(1.04–1.67)**
Yes (Ref)	4.7% (2.9–6.4)	(Ref)	(Ref)	31.9% (28.2–35.6)	(Ref)	(Ref)
Lives alone						
Yes	14.0% ^d^	**2.23**	0.90	41.1%	**1.30**	0.98
	(9.0–19.0)	**(1.39–3.57)**	(0.52–1.55)	(33.7–48.5)	**(1.05–1.61)**	(0.77–1.25)
No (Ref)	6.5%	(Ref)	(Ref)	34.0%	(Ref)	(Ref)
	(4.6–8.4)			(30.5–37.6)		

Ref: Reference level; All percentages and aPRs are based on weighted survey data.

aPR: Adjusted prevalence ratio derived from modified log-Poisson regression model; CI: Confidence interval.

^a^ Bivariate aPR is a comparison of the prevalence of the outcome at the comparator level vs. reference level after adjusting for sex, race/ethnicity, and age (10-year interval variable) based on results of modified log-Poisson regression models; aPRs with a 95% CI that does not include 1.00 (bolded text) are statistically significant at p < .05.

^b^ Full model aPR is a comparison of the prevalence of the outcome at the comparator level vs. reference level after adjusting for sex, race/ethnicity, and age, and the other social connectedness risk variables based on results of modified log-Poisson regression models. aPRs with a 95% CI that does not include 1.00 (bolded text) are statistically significant at p < .05.

^c^ How often sees or talks to people cares about or feels close to? (For example, talking to friends on the phone, visiting friends or family, going to church or club meetings).

^d^ Significantly different from reference level at p < .05.

^e^ Single, separated, or widowed vs. married, living with partner, or in a committed relationship but not living together

The prevalence of SOCSUPT risk also decreased monotonically as SOCCON frequency increased (70.2%, 48.1%, 29.3%, and 15.9%, respectively). **Table 2** shows that compared to a SOCCON frequency of ≥ 5 days/week, the prevalence of SOCSUPT risk was more than four times higher for those with SOCCON frequency of <1 day/week (aPR = 4.32 [3.27–5.70]), nearly three times higher for contacts on 1–2 days/week (aPR = 2.90 [2.17–3.86]), and 70% higher for contacts on 3–4 days/week (aPR = 1.77 [1.28–2.45]). Not shown is that the prevalence of SOCSUPT risk significantly differed across various levels of SOCCON frequency. Individuals with the lowest level of SOCCON frequency (< 1 day/week) had a 60% higher prevalence of SOCSUPT risk compared to those with contacts 1–2 days/week (aPR = 1.49 [1.25–1.77]. Similarly, individuals with social contacts 1–2 days/week had a 60% higher prevalence of SOCSUPT risk compared to those with contacts 3–4 days/week (aPR = 1.63 [1.28–2.09. In the full model predicting SOCSUPT risk, controlling for LONELINESS, NO_RELAT, and LIVALONE risks only slightly affected the association of SOCCON frequency with prevalence of SOCSUPT risk at the 1–2 days/week and 3–4 days/week levels, but had a somewhat larger effect on the comparison of < 1 day/week vs. ≥ 5 days/week. After adjusting for other factors, NO_RELAT risk was associated with a 32% increase in prevalence of SOCSUPT risk (aPR = 1.32 [1.04–1.67]), but LIVALONE risk was no longer statistically significant. Finally, while in the full model LONELINESS risk nearly doubled prevalence of SOCSUPT risk (aPR = 1.94 [1.63–2.31]), the magnitude of this association was much smaller than that of the association of SOCSUPT risk with LONELINESS risk.

### Association of social connectedness factors with emotional health outcome measures

The independent cross-sectional associations of the five SC risks with the three emotional health variables are shown in **Table 3**. In the unadjusted bivariate comparisons (see columns with “%” heading), SOCSUPT risk, LONELINESS risk, and SOCCON frequency of < 1 day/week vs. ≥ 5 days/week all significantly increased prevalence of being emotionally troubled, having fair/poor emotional health, and being chronically stressed. NO_RELAT risk was only significantly associated with having higher prevalence of fair/poor emotional health, and LIVALONE risk was not significantly associated with any of the emotional health status variables.

**Table 3:** Associations of the five social connectedness risk variables with emotional health measures.

Social connectedness variable	Quite emotionally troubled during past 4 weeks ^a^			Fair/poor emotional health ^b^			Chronic high stress ^c^		
	%	Bivariate model ^d^ aPR (95% CI)	Full model ^e^ aPR (95% CI)	%	Bivariate model ^d^ aPR (95% CI)	Full model ^e^ aPR (95% CI)	%	Bivariate model ^d^ aPR (95% CI)	Full model ^e^ aPR (95% CI)
Social connection frequency ^f^									
< Once a week	26.0% ^g^ (17.8–34.2)	**2.26** **(1.44–3.52)**	0.86 (0.54–1.39)	23.6% ^g^ (15.8–31.5)	**2.93** **(1.74–4.93)**	1.01 (0.58–1.76)	26.5%^g^ (18.3–34.7)	**1.79** **(1.17–2.73)**	0.79 (0.50–1.24)
1–2 times/week	18.4% (13.4–23.5)	1.41 (0.93–2.15)	0.79 (0.53–1.17)	15.2% ^g^ (10.5–19.9)	**1.77** **(1.07–2.93)**	0.89 (0.56–1.39)	19.0% (13.6–24.4)	1.17 (0.79–1.75)	0.73 (0.48–1.10)
3–4 times/week	11.0% (6.9–15.1)	0.85 (0.52–1.39)	0.67 (0.44–1.02)	9.9% (5.9–13.9)	1.13 (0.64–2.00)	0.86 (0.52–1.40)	14.2% (9.0–19.4)	0.87 (0.55–1.38)	0.74 (0.48–1.13)
≥ 5 times/week (Ref)	12.4% (8.4–16.3%)	(Ref)	(Ref)	8.3% (5.0–11.6)	(Ref)	(Ref)	14.4% (10.2–18.6)	(Ref)	(Ref)
How often gets enough social and emotional support									
Never, rarely, or sometimes	31.2% ^g^ (25.8–36.6)	**4.27** **(3.01–6.06)**	**3.67** **(2.50–5.40)**	27.1% ^g^ (22.1–32.1)	**5.49** **(3.50–8.64)**	**3.85** **(2.29–6.47)**	32.1%^g^ (26.6–37.5)	**3.17** **(2.27–4.43)**	**2.93** **(2.04–4.23)**
Usually/always (Ref)	7.0% (4.9–9.1)	(Ref)	(Ref)	4.8% (2.9–6.7)	(Ref)	(Ref)	9.1% (6.5–11.8)	(Ref)	(Ref)
How often feels lonely or socially isolated									
Often/always (Ref)	53.4% ^g^ (41.2–65.7)	**4.09** **(2.99–5.59)**	**2.24** **(1.52–3.31)**	56.6% ^g^ (44.3–68.9)	**6.14** **(4.44–8.57)**	**2.86** **(1.91–4.29)**	49.4%^g^ (37.2–61.6)	**3.12** **(2.23–4.37)**	**1.99** **(1.34–2.95)**
Never, rarely, sometimes	12.3% (9.9–14.7)	(Ref)	(Ref)	9.0% (7.0–11.1)	(Ref)	(Ref)	14.5% (11.9–17.2)	(Ref)	(Ref)
In a committed relationship ^h^									
No	19.1 (13.7–24.5)	1.30 (0.91–1.86)	1.00 (0.64–1.57)	20.4% ^g^ (14.9–25.9)	**2.11** **(1.45–3.06)**	1.55 (0.91–2.63)	18.4% (13.0–23.9)	1.12 (0.78–1.61)	0.90 (0.58–1.37)
Yes (Ref)	14.3 (11.5–17.1)	(Ref)	(Ref)	10.0% (7.7–12.4)	(Ref)	(Ref)	16.9% (13.8–20.0)	(Ref)	(Ref)
Lives alone									
Yes	14.1%	0.95	0.80	15.0%	1.31	0.76	13.5%	0.87	0.82
	(9.3–19.0)	(0.64–1.41)	(0.50–1.27)	(10.1–19.8)	(0.90–1.92)	(0.47–1.20)	8.8–19.3)	(0.58–1.30)	(0.51–1.31)
No (Ref)	15.8%	(Ref)	(Ref)	12.2%	(Ref)	(Ref)	18.0%	(Ref)	(Ref)
	(13.0–18.6)			(9.7–14.8)			(14.9–21.0)		

Ref: Reference level; All percentages and aPRs are based on weighted survey data.

aPR: Adjusted prevalence ratio; CI: Confidence interval

^a^ During the past four weeks, was bothered quite a bit/extremely (vs. not at all, a little, or somewhat) by emotional troubles such as feeling anxious, irritable, depressed or sad

^b^ In general, rates overall mental or emotional health as fair or poor (vs. good, very good, or excellent).

^c^ During the past 3 months felt very stressed or tense much or most of the time (vs. never, a little of the time, or some of the time).

^d^ Bivariate aPR compares prevalence of outcome in comparator level vs. reference level after adjusting for sex, race/ethnicity, and age (10-year interval variable) derived from modified log-Poisson regression models with weighted survey data; aPRs with a 95% CI that does not include 1.00 (bolded text) are statistically significant at p < .05.

^e^ Full model aPR compares prevalence of outcome in comparator level vs. reference level after adjusting for sex, race/ethnicity, and age, and the other social connectedness risk variables. aPRs with a 95% CI that does not include 1.00 (bolded text) are statistically significant at p < .05.

^f^ How often sees or talks to people cares about or feels close to? (For example, talking to friends on the phone, visiting friends or family, going to church or club meetings)

^g^ Significantly different from reference level at p < .001.

^h^ Single, separated, or widowed vs. married, living with partner, or in a committed relationship but not living together.

The demographics-adjusted model of being emotionally troubled (see column with “Bivariate” heading) shows a four-fold increase in prevalence of being emotionally troubled among those with SOCSUPT risk (aPR = 4.27 [3.01–6.06]) and LONELINESS risk (aPR = 4.09 [2.99–5.59]), but no significant difference was seen for NO_RELAT and LIVALONE risks. Adults with the lowest versus highest level of SOCCON frequency (<1 day/week vs. ≥ 5 days/week) had a two-fold higher prevalence of being emotionally troubled (aPR = 2.26 [1.44–3.52]), but those with higher frequencies of social contacts did not differ from those with contacts ≥ 5 days/week. In the full model that included all five SC variables, only SOCSUPT risk (aPR = 3.67 [2.50–5.40]) and LONELINESS risk (aPR = 2.24 [1.52–3.31]) remained independently associated with being emotionally troubled.

Prevalence of fair/poor emotional health was nearly three times higher among those with a SOCCON frequency of <1 day/week (aPR = 2.93 [1.74–4.93]) and about 70% higher among those with SOCCON frequency of 1–2 days/week (aPR = 1.77 [1.07–2.93]) compared to the ≥ 5 days/week level, with no difference between the 3–4 days/week vs. ≥ 5 days/week levels. Both LONELINESS risk (aPR = 6.14 [4.44–8.57]) and SOCSUPT risk (aPR = 5.49 [3.50–8.64]) were associated with approximately six-fold higher prevalence of fair/poor emotional health, and NO_RELAT was associated with a two-fold increased prevalence (aPR = 2.11 [1.45–3.06]), but LIVALONE was not significantly associated. In the full model, only SOCSUPT risk (aPR = 3.85 [2.29–6.57]) and LONELINESS risk (aPR = 2.86 [1.91–4.29]) remained significant independent predictors of having fair/poor emotional health.

A three-fold increase in the prevalence of chronic stress was seen for those with SOCSUPT risk (aPR = 3.17 [2.27–4.43]) and LONELINESS risk (aPR = 3.12 [2.23–4.37]). A SOCCON frequency of < 1 day/week vs. ≥ 5 days/week was associated with an 80% higher prevalence (aPR = 1.79 [1.17–2.73]), but significant differences were not seen for the two higher SOCCON frequencies. No significant association with chronic stress was seen for NO_RELAT or LIVALONE risks. In the full model, only SOCSUPT risk (aPR = 2.93 [2.04–4.23]) and LONELINESS risk (aPR = 1.99 [1.34–2.95]) remained significant independent predictors of being chronically stressed.

The AUCs for models that included the SOCCON frequency, LONELINESS risk, and SOCSUPT risk variables included in the proposed 3-item Brief Social Connected Screener were 0.73 for being emotionally troubled, 0.76 for fair/poor emotional health, and 0.74 for being chronically stressed. The models of these outcomes that included only LONELINESS risk and SOCSUPT risk had AUCs very close to those that also included SOCCON frequency (0.72, 0.72, 0.72, respectively). Finally, the models that included all five SC risks had AUCs very similar to those for the three variable models (0.73, 0.77, 0.75, respectively). For reference, AUCs in the of .70-.79 range are considered a good, but not strong, model fit.

The sensitivity analyses by age group showed that the same pattern of associations of social connectedness factors observed in the full sample were seen in the middle-aged and older aged subgroups. However, LONELINESS risk was more strongly associated with perceived lack of adequate social support and all three emotional health outcomes in the older versus middle-aged subgroup **(See S1 and S2 Tables in S2 Appendix)**. The AUCs for the models also did not meaningfully differ by age group.

## Discussion

Using data from a health plan member survey conducted in 2021, approximately one year following the onset of the COVID-19 emergency, we examined the inter-relationship of variables representing three subdomains risks of social connectedness (frequency of social connections, frequent feelings of loneliness/feelings of socially isolation, and feelings of not usually getting enough social/emotional support). These subdomains were identified in the IOM SBDoH Subcommittee report as important factors to screen for and capture in electronic health records, along with two structural social connectedness risk factors, living alone and not being in a committed relationship. We then further examined the association of these five social connectedness risk variables with three single-item patient-reported measures of emotional distress.

**Fig 1:**
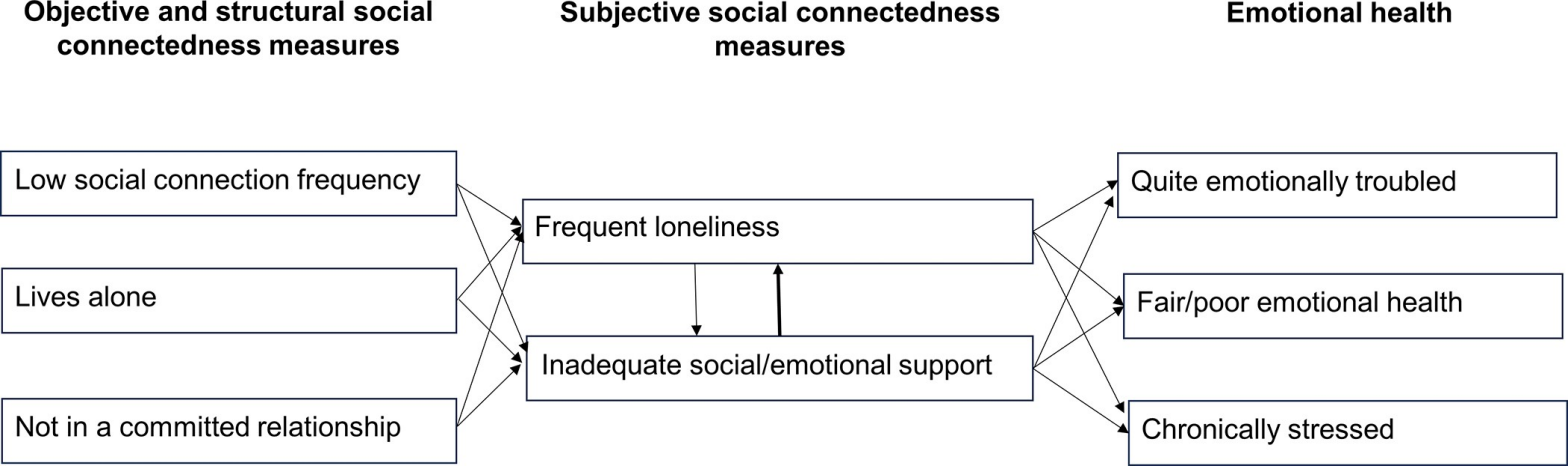
Flow diagram showing inter-relationships of objective, structural, and subjective social connectedness risks and relationships of social connectedness risks with emotional health outcomes. (1) Low frequency of social contact with others one cares about and feels close to, living alone, and not being in a committed relationship all increase risks of frequent loneliness and perception of inadequate social/emotional support. (2) Perception of inadequate social/emotional support and frequent loneliness are inter-related, but perception of inadequate social support has a much stronger influence on risk of frequent loneliness. (3) Frequent loneliness and perception of inadequate social/emotional support risks, but not objective and structural risks, are directly associated with increased risk of having the three negative emotional health states.

We found that frequency of social contacts (seeing or talking) with people the individual cares about or feels close to had a significant direct effect on the two subjective SC risks (frequently feeling lonely/socially isolated and perceiving inadequate social/emotional support), especially if those social contacts were occurring less than 3 days/week. The two structural SC risks, living alone and not being in a committed relationship, were also significantly associated with the subjective SC risks. However, in the full models that also included the two subjective SC risks, the objective and structural SC risks were not significantly associated with the emotional health outcomes, nor did their inclusion in the full models improve accuracy for classifying adults as having the emotional troubles and chronic stress compared to models that only included the two subjective SC risks. This finding is in line with other authors who have suggested that the association of objective and structural SC risks with emotional well-being is mediated through the much stronger relationships between loneliness/feelings of social isolation and perceived inadequacy of social support [59, 60] (See **Fig 1**).

Additionally, we found that inadequate social support had a much larger impact on loneliness than any of the three objective/structural SC risks, and that the magnitude of the effect of loneliness on inadequate social/emotional support was much smaller than the magnitude of the effect of inadequate social support on loneliness. Zhang and Dong, based on the results of a meta-analysis and drawing on cognitive and interaction theories, proposed that perception of the adequacy of social support received from one’s social network mediates the association between the amount and types of social connections an individual has and loneliness [44]. They suggest that intervening to increase an individual’s level of social and emotional support and/or increasing the individual’s perception of the adequacy of the social support they are receiving may be a more effective approach to preventing or remediating loneliness than simply trying to enlarge an individual’s social network or increase frequency of their social contacts.

Individuals can vary, based on their personality and situational circumstances, regarding the amount and types of social contact they need and want with close friends, family, and intimate partners, as well as the nature of social/emotional support they expect from different people in their social network. Also, while extensive research has shown that people who get sufficient social and emotional support from their spouses, family members, and friends have better physical and mental health than those who do not [61], there is increasing research showing that frequent unsupportive or negative social interactions may have a detrimental effect on physical and mental health [62, 63]. For example, individuals can feel overwhelmed, undermined, and stressed if they perceive that they are being too overly monitored or are getting undesired social or emotional support from these interactions [64]. Individuals in spousal or other domestic relationships that are psychologically dysfunctional, that have high amounts of negative interactions, or that are not satisfying their emotional and/or social needs or expectations can be at higher risk for depression, stress, and loneliness or feelings of social isolation than if they were not in that relationship [62].

In the context of previous research on the relationship of social connectedness and health and mental health, our study results suggest that if the goal is to identify people who have or are at risk for developing poor emotional health, cognitive decline, and poor health outcomes, having patient-reported information about perceived adequacy of social/emotional support they are getting and frequency of feeling lonely or socially isolated may be more important than knowing about frequency of social contacts and whether the individual is not in a committed relationship or lives alone. The proposed 3-item Brief Social Connectedness Screener covers all three subdomains of social connectedness (social integration/isolation, social support, and loneliness) that the IOM subcommittee on SBDoH stated would help healthcare providers better understand patient health and use of and need for health care services, as well as to identify at-risk patients and to intervene at a patient or population level [5].

We are not suggesting that the more commonly used questions to ascertain frequency of social contacts, committed relationship status, and living situation are not valuable to include as part of social connectedness screening. Knowing that an individual has a low weekly frequency of connecting with people they feel close to and that they are not in a committed relationship could help identify people at elevated risk for becoming very lonely or feeling that they are not getting adequate social and emotional support from their social network. People who feel with low frequency of social contacts with people they feel close to and feel that they are not getting enough emotional support from their current social network can be encouraged to spread their contacts with people they care about throughout the week and to develop meaningful relationships with more people in order to increase potential opportunities for supportive social interactions. Knowing whether an individual lives alone, has no significant other, and/or is frequently connecting with other people who know and care about them can also help identify individuals at risk for having physical or emotional decline go unnoticed or for lacking help with activities of daily living if needed.

In this study, an estimated 40% of adults were connecting with close family and friends <3 times/week. This not surprising, given that the survey data used for this study were collected approximately one year into the COVID-19 emergency, when people in California were just beginning to socialize with people outside their “household bubbles” due to the wider availability of face masks, vaccines, and “COVID-friendly” venues. However, there are many other circumstances in which in-person contact with close friends and family may be limited, including having a highly contagious disease, suppressed immunity due to cancer treatment, caregiving responsibilities, or loss of physical proximity to friends and family due to travel, a move, or job change. Fortunately, low frequency of social connection with close friends and family, inadequate social/emotional support, and frequent feelings of loneliness/social isolation are all modifiable social connectedness risks amenable to psychoeducation and cognitive interventions [65, 66].

Our study had several strengths, including the large sociodemographically diverse study population, use of validated social connectedness screening questions, and the fact that the survey data were collected during a time when, due to the COVID-19 emergency, in-person social contacts were limited, and prevalence of loneliness and feelings of social isolation was increasing in the population. However, we acknowledge study limitations that may affect generalizability of the results, including a 29% survey response rate, a sample representative of one Northern California health plan, exclusion of non-English speakers and adults who could not be reached by mail, and the collection of survey data early in the second year of the COVID-19 emergency, which likely had a large effect on responses to the social connection frequency and loneliness/social isolation questions. Another limitation is that at least some people may have misinterpreted the social connection question as only referring to connections with people outside of their immediate household, as evidenced by the lack of difference in reported social connection frequency between those in and not in a committed relationship status and by those living alone versus with other people. The relatively small number of adults who lived alone (16%), of whom 12% were in a committed relationship, likely limited our statistical power to test for the independent association of living alone with the three social connectedness screening measures and three emotional health outcomes. A further limitation is our use of single-item self-reported mental/emotional health (SRMH) measures to validate our social connectedness subdomains. In a scoping review, Ahmad *et al*. found that single-item measures of SRMH were only moderately correlated with multi-item mental health scales. However, they suggest that single-item SRMH measures, like single-item measures of self-reported overall health, may eventually be found to be equally or stronger predictors of mortality, morbidity, and healthcare utilization than many commonly used measures, although this may vary cross-culturally [67]. Finally, while research suggests that social connectedness risks can adversely affect physical, cognitive, and mental health, we were limited by our dataset with regard to examining the association of these social connectedness risks with additional clinical health and mental health outcomes.

The three questions that we are recommending for use to screen adults for subdomain risks in the broader social connectedness domain come from a widely used social risk screening questionnaire and national health surveys fielded with diverse populations in multiple languages. However, further research is needed to evaluate the psychometric properties of these screening items when used in a healthcare setting, as well as whether there are cross-cultural and sociodemographic group differences in how these questions are interpreted and how the social connectedness risks are related to physical, emotional, and mental health disorders.

## Conclusions

In this study conducted approximately one year into the COVID-19 emergency, we found that a single-item measure of low frequency social contacts with close friends and family was significantly associated with single-item measures of feelings of loneliness/social isolation and perceived social/emotional support, but not with single-item patient-reported measures of emotional distress, fair or poor emotional/mental health, and chronic high levels of stress. However, the subjective items measuring frequent feelings of loneliness/social isolation and perceived inadequacy of social/emotional support were significantly associated with all three emotional health measures. We believe that our results provide evidence that screening for social connectedness risk could be improved by including questions about subjective experience of loneliness/social isolation and social/emotional support in addition to frequency of social connections and demonstrate that this can be done without substantially adding to the length of social risk screening tools.

## Supporting information

S1 Appendix Berkman-Syme Social Connection Index and scoring. (DOCX)

S2 Appendix (DOCX)

S1 Dataset URL for published study dataset (PONE-D-23-40406). (DOCX)
