# Supplementary material for: A brief but comprehensive three-item social connectedness screener for use in social risk assessment tools
Source: PLoS One. 2024 Jul 19;19(7):e0307107. doi: 10.1371/journal.pone.0307107 (PMC11259274; doi:10.1371/journal.pone.0307107)
Supplement: S1 Appendix — (DOCX) [file pone.0307107.s001.docx]

**Appendix A**

**Berkman-Syme Social Connection Index (B-S SCI) recommended by IOM Subcommittee on Social and Behavioral Determinants of Health for use in Electronic Health Records**

1. In a typical week, how many times do you talk on the phone with family, friends, or neighbors?

[Never, Once a week, Twice a week, Three times a week, More than three times a week]

2. How often do you get together with friends or relatives?

[Never, Once a week, Twice a week, Three times a week, More than three times a week]

3. How often do you attend church or religious services?

[Never, 1 to 4 times per year, More than 4 times per year]

4. Do you belong to any clubs or organizations such as church groups, unions, fraternal or athletic groups, or school groups?

[Yes, No]

5. How often do you attend meetings of the clubs or organizations you belong to?^1^

[Never, 1 to 4 times per year, More than 4 times per year]

6. Are you married, widowed, divorced, separated, never married, or living with a partner?

[Married, Widowed, Divorced, Separated, Never married, Living with partner]

Risk scoring algorithm ^1^:

Patients receive 1 point for each of the following:

- being married or living together with someone in a partnership at the time of questioning
- averaging 3 or more interactions per week with other people (assessed with the questions "In a typical week, how many times do you talk on the telephone with family, friends, or neighbors?" and "How often do you get together with friends or relatives?")
- attending church or religious services 4 or more times per year
- participating in a club or organization such as a church group, union, fraternal or athletic group, or school group

Scores range from 0 to 4, with 0 representing the highest level of social isolation and 4 representing the lowest level. However, individuals with a score of 0 or 1 are considered very socially isolated.

^1^ Note: The items in this version of the B-S SCI come from the Third National Health and Nutrition Examination Survey conducted 1998-2004. In the NHANES questionnaires, question 5 (frequency of attending club/organization meetings) was a follow-up question asked of people who answered “Yes” to question 4. This suggests that the intention of those who created the NHANES B-S SCI scale was that frequency of participation in clubs and organizations would be scored based on both questions 4 and 5, with a “No” response to question 4 or responses of “Never” or 1-4 times a year” to question 5 indicating no or low club/organization attendance. This would have made the scoring of club/organization participation parallel that of the question about frequency of attending religious services. Using the current scoring algorithm, information from question 5 is not used in the scoring, and someone who belongs to a club or organization but never or rarely attends meetings (in person or virtually) still earns a point for that type of social connection.

Sources for risk scoring:

Pantell M, Rehkopf D, Jutte D, Syme SL, Balmes J, Adler N. Social isolation: a predictor of mortality comparable to traditional clinical risk factors. Am J Public Health. 2013 Nov;103(11):2056-62. doi: 10.2105/AJPH.2013.301261.

Ford ES, Loucks EB, Berkman LF. Social integration and concentrations of C-reactive protein among US adults. Ann Epidemiol. 2006 Feb;16(2):78-84. doi: 10.1016/j.annepidem.2005.08.005
