# Supplementary material for: A brief but comprehensive three-item social connectedness screener for use in social risk assessment tools
Source: PLoS One. 2024 Jul 19;19(7):e0307107. doi: 10.1371/journal.pone.0307107 (PMC11259274; doi:10.1371/journal.pone.0307107)
Supplement: S2 Appendix — (DOCX) [file pone.0307107.s002.docx]

| **APPENDIX B**  **Table S1. Inter-relationship of five social connectedness variables, by age group** | | | | | |  |
| --- | --- | --- | --- | --- | --- | --- |
| **Variable** | **Age group** | **Often feels lonely or socially isolated**  **(Loneliness risk)** | | **Usually does not get enough social and emotional support**  **(SOCSUPT risk)** | | |
|  |  | **Bivariate model ^a^**  **aPR (95% CI)** | **Full model ^b^**  **aPR (95% CI)** | **Bivariate model ^a^**  **aPR (95% CI)** | **Full model ^b^**  **aPR (95% CI)** | |
| Social connection frequency ^c^  (Ref: ≥ 5 times/week) |  |  |  |  |  | |
| < Once a week | 35-64 yr | **9.44 (3.09-28.83)** | **3.48 (1.03-11.7)** | **3.96 (2.78-5.63)** | **3.39 (2.35-4.91)** | |
|  | 65-85 yr | **8.37 (3.73-8.76)** | 2.05 (0.91-4.63) | **5.12 (3.70-7.07)** | **4.01 (2.87-5.61)** | |
| 1-2 times/week | 35-64 yr | **3.80 (1.25-11.53)** | 1.72 (0.53-5.58) | **2.71 (1.88-3.91)** | **2.55 (1.77-3.67)** | |
|  | 65-85 yr | **4.76 (2.14-10.62)** | 1.73 (0.79-3.77) | **3.52 (2.55-4.86)** | **3.08 (2.23-4.25)** | |
| 3-4 times/week | 35-64 yr | 1.64 (0.41-6.46) | 1.20 (0.30-4.78) | **1.55 (1.01-2.39)** | **1.55 (1.02-2.35)** | |
|  | 65-85 yr | 2.10 (0.87-5.07) | 0.89 (0.37-2.13) | **2.57 (1.83-3.60)** | **2.46 (1.76-3.43)** | |
|  |  |  |  |  |  | |
| Gets enough social and emotional support  Never, rarely, or sometimes vs. Usually/always |  |  |  |  |  | |
|  |  |  |  |  |  | |
|  | 35-64 yr | **33.27 (9.49-116.66)** | **20.13 (5.93-68.32)** |  |  | |
|  | 65-85 yr | **16.40 (8.13-33.10)** | **11.55 (5.51-24.22)** |  |  | |
|  |  |  |  |  |  | |
| Frequency feels lonely/socially isolated: |  |  |  |  |  | |
| Often/always vs. Never, rarely, or sometimes | 35-64 yr |  |  | **2.76 (2.35-3.23)** | **1.79 (1.44-2.24)** | |
|  | 65-85 yr |  |  | **3.76 (3.17-4.45)** **^d^** | **2.52 (2.08-3.07)** **^d^** | |
|  |  |  |  |  |  | |
| In a committed relationship^e^:  No vs. Yes | 35-64 yr | **3.36 (1.76-3.43)** | **2.96 (1.41-6.20)** | **1.48 (1.15-1.90)** | **1.36 (1.01-1.83)** | |
|  | 65-85 yr | **4.18 (2.46-7.12)** | **2.26 (1.25-4.08)** | **1.56 (1.26-1.93)** | **1.28 (1.00-1.64)** | |
|  |  |  |  |  |  | |
| Lives alone:  Yes vs. No | 35-64 yr | **2.03 (1.00-4.09)** | 0.81 (0.37-1.76) | 1.29 (0.97-1.74) | 0.98 (0.72-1.33) | |
|  | 65-85 yr | **2.82 (1.69-4.68)** | 1.27 (0.74-2.17) | **1.34 (1.06-1.68)** | 0.95 (0.73-1.22) | |
| Ref: Reference level; All percentages and aPRs are based on weighted survey data.  aPR: Adjusted prevalence ratio derived from modified log-Poisson regression model; CI: Confidence interval  ^a^ Bivariate aPR is a comparison of the prevalence of the outcome at the comparator level vs. reference level after adjusting for sex, race/ethnicity, and age group (10 yr intervals) based on results of modified log-Poisson regression models; aPRs with a 95% CI that does not include 1.00 (bolded font) are statistically significant at p<.05.  ^b^ Full model aPR is a comparison of the prevalence of the outcome at the comparator level vs. reference level after adjusting for sex, race/ethnicity, age group (10 yr intervals), and the other social connectedness risk variables based on results of modified log-Poisson regression models; aPRs with a 95% CI that does not include 1.00 (bolded font) are statistically significant at p<.05.  ^c^ How often sees or talks to people cares about or feels close to? (For example, talking to friends on the phone, visiting friends or family, going to church or club meetings).  ^d^ Difference in aPRs for this factor between 35-64 and 65-85 yr age groups is statistically significant at p<.05.  ^e^ Single, separated, or widowed vs. married, living with partner, or in a committed relationship but not living together | | | | | |  |

| **Table S2. Associations of the five social connectedness risk variables with emotional health measures, by age group** | | | | | | | | |  |
| --- | --- | --- | --- | --- | --- | --- | --- | --- | --- |
| **Social connectedness variable** | **Age group** | **Quite emotionally troubled during past 4 weeks ^a^** | | **Fair/poor emotional health ^b^** | | **Chronic high stress ^c^** | | | |
|  |  | **Bivariate model ^d^**  **aPR (95% CI)** | **Full model ^e^**  **aPR (95% CI)** | **Bivariate model ^d^**  **aPR (95% CI)** | **Full model ^e^**  **aPR (95% CI)** | **Bivariate model ^d^**  **aPR (95% CI)** | **Full model ^e^**  **aPR (95% CI)** | | |
| Social connection frequency ^f^  Ref: ≥ 5 times/week |  |  |  |  |  |  |  | | |
| < Once a week | 35-64 yr | **2.05 (1.18-3.56)** | 0.83 (0.47-1.49) | **2.60 (1.30-5.19)** | 0.96 (0.45-2.06) | 1.62 (0.99-2.66) | 0.75 (0.45-1.26) | | |
|  | 65-85 yr | **2.96 (1.74-5.05)** | 1.10 (0.62-1.95) | **3.97 (2.37-6.66)** | 1.20 (0.70-2.07) | **2.79 (1.56-5.00)** | 1.02 (0.52-2.01) | | |
| 1-2 times/week | 35-64 yr | **1.33 (0.79-2.22)** | 0.76 (0.47-1.23) | 1.61 (0.82-3.16) | 0.86 (0.42-1.75) | 1.05 (0.66-1.70) | 0.67 (0.42-1.0) | | |
|  | 65-85 yr | **1.78 (1.10-2.90)** | 0.93 (0.56-1.52) | **2.16 (1.31-359)** | 0.95 (0.60-1.49) | **1.96 (1.17-3.28)** | 1.02 (0.61-1.71) | | |
| 3-4 times/week | 35-64 yr | 0.77 (0.41-1.46) | 0.63 (0.36-1.10) | 0.98 (0.44-2.16) | 0.80 (0.41-1.59) | 0.87 (0.52-1.48) | 0.75 (0.46-1.23) | | |
|  | 65-85 yr | 1.11 (0.66-1.87) | 0.80 (0.49-1.32) | 1.46 (0.87-2.45) | 0.93 (0.56-1.55) | 0.82 (0.84-1.51) | 0.58 (0.32-1.05) | | |
|  |  |  |  |  |  |  |  | | |
| Gets enough social/ emotional support : |  |  |  |  |  |  |  | | |
| Never, rarely, or  Sometimes vs.  Usually/always | 35-64 yr | **4.23 (2.67-6.69)** | **3.88 (2.37-6.36)** | **5.49 (2.88-10.49)** | **4.09 (1.96-8.52)** | **2.95 (2.00-4.36)** | **2.94 (1.93-4.49)** | | |
|  | 65-85 yr | **4.30 (2.99-6.16)** | **2.54 (1.60-4.04) ^g^** | **5.94 (4.09-8.62)** | **3.45 (2.19-5.43)** | **4.42 (2.94-6.65)** ^g^ | **2.84 (1.71-4.71)** | | |
|  |  |  |  |  |  |  |  | | |
| Frequency feels lonely/socially isolated |  |  |  |  |  |  |  | | |
| Often/always vs. Never, rarely, or sometimes | 35-64 yr | **3.36 (2.25-5.02)** | **1.85 (1.13-3.02)** | **5.20 (3.34-.09)** | **2.41 (1.38-4.20)** | **2.56 (1.71-3.85)** | **1.69 (1.05-2.71)** | | |
|  | 65-85 yr | **8.01 (5.81-11.02)** **^g^** | **4.84 (3.10-7.55) ^g^** | **9.34 (6.95-12.57) ^g^** | **4.33 (2.89-6.51) ^g^** | **7.00 (4.82-10.18) ^g^** | **3.82 (2.25-6.51) ^g^** | | |
|  |  |  |  |  |  |  |  | | |
| In a committed relationship ^h^ |  |  |  |  |  |  |  | | |
| No vs. Yes | 35-64 yr | 1.35 (0.85-2.14) | 1.08 (0.63-1.84) | **2.21 (1.33-3.67)** | 1.67 (0.83-3.37) | 1.09 (0.70-1.70) | 0.89 (0.53-1.47) | | |
|  | 65-85 yr | 1.25 (0.83-1.86) | 0.80 (0.46-1.41) | **1.92 (1.31-2.81)** | 1.25 (0.80-1.94) | 1.27 (0.82-1.96) | 0.94 (0.53-1.67) | | |
|  |  |  |  |  |  |  |  | | |
| Lives alone |  |  |  |  |  |  |  | | |
| Yes vs. No | 35-64 yr | 0.89 (0.51-1.57) | 0.72 (0.39-1.33) | 1.23 (0.68-2.23) | 0.72 (0.37-1.37) | 0.81 (0.47-1.38) | 0.78 (0.43-1.42) | | |
|  | 65-85 yr | 1.11 (0.72-1.73) | 0.99 (0.54-1.80) | 1.44 (0.97-2.13) | 0.86 (0.53-1.37) | 1.01 (0.62-1.64) | 0.84 (0.46-1.53) | | |
| Ref: Reference level; All percentages and aPRs are based on weighted survey data.  aPR: Adjusted prevalence ratio; CI: Confidence interval.  ^a^ During the past four weeks, was bothered quite a bit/extremely (vs. not at all, a little, or somewhat) by emotional troubles such as feeling anxious, irritable, depressed or sad  ^b^ In general, rates overall mental or emotional health as fair or poor (vs. good, very good, or excellent).  ^c^ During the past 3 months felt very stressed or tense much or most of the time (vs. never, a little of the time, or some of the time).  ^d^ Bivariate aPR compares prevalence of outcome in comparator level vs. reference level after adjusting for sex, race/ethnicity, and age (10-year interval variable) derived from modified log-Poisson regression models; aPRs with a 95% CI that does not include 1.00 (bolded font) are statistically significant at p<.05.  ^e^ Full model aPR compares prevalence of outcome in comparator level vs. reference level after adjusting for sex, race/ethnicity, and age, and the other social connectedness risk variables; aPRs with a 95% CI that does not include 1.00 (bolded font) are statistically significant at p<.05.  ^f^ How often sees or talks to people cares about or feels close to? (For example, talking to friends on the phone, visiting friends or family, going to church or club meetings)  ^g^ Difference in aPRs for this factor between 35-64 and 65-85 yr age groups is statistically significant at p<.05.  ^h^ Single, separated, or widowed vs. married, living with partner, or in a committed relationship but not living together. | | | | | | | |  |  |
