# Supplementary material for: A brief but comprehensive three-item social connectedness screener for use in social risk assessment tools
Source: PLoS One. 2024 Jul 19;19(7):e0307107. doi: 10.1371/journal.pone.0307107 (PMC11259274; doi:10.1371/journal.pone.0307107)
Supplement: S1 Dataset — (DOCX) [file pone.0307107.s003.docx]

The published study dataset used for PONE-D-23-40406 “A brief but comprehensive three-item social connectedness screener for use in social risk assessment tools” can be found at:

Gordon, Nancy, 2024, "Replication Data for: A brief but comprehensive three-item social connectedness screener for use in social risk assessment tools (PLOS One article)", <https://doi.org/10.7910/DVN/LGTQWR>, Harvard Dataverse, V1
